# Osteopathy modulates brain–heart interaction in chronic pain patients: an ASL study

**DOI:** 10.1038/s41598-021-83893-8

**Published:** 2021-02-25

**Authors:** Francesco Cerritelli, Piero Chiacchiaretta, Francesco Gambi, Raoul Saggini, Mauro Gianni Perrucci, Antonio Ferretti

**Affiliations:** 1 Department of Neuroscience, Imaging and Clinical Sciences, “G. D’Annunzio” University of Chieti-Pescara, Via dei Vestini, 33, Chieti Scalo, Italy; 2 ITAB-Institute for Advanced Biomedical Technologies, “G. D’Annunzio” University of Chieti-Pescara, Chieti, Italy; 3 Clinical-Based Human Research Department, Foundation C.O.ME. Collaboration, Pescara, Italy; 4 School of Specialty in Physical and Rehabilitation Medicine, “G. D’Annunzio” University of Chieti-Pescara, Chieti, Italy

**Keywords:** Neuroscience, Health care

## Abstract

In this study we used a combination of measures including regional cerebral blood flow (rCBF) and heart rate variability (HRV) to investigate brain–heart correlates of longitudinal baseline changes of chronic low back pain (cLBP) after osteopathic manipulative treatment (OMT). Thirty-two right-handed patients were randomised and divided into 4 weekly session of OMT (N = 16) or Sham (N = 16). Participants aged 42.3 ± 7.3 (M/F: 20/12) with cLBP (duration: 14.6 ± 8.0 m). At the end of the study, patients receiving OMT showed decreased baseline rCBF within several regions belonging to the pain matrix (left posterior insula, left anterior cingulate cortex, left thalamus), sensory regions (left superior parietal lobe), middle frontal lobe and left cuneus. Conversely, rCBF was increased in right anterior insula, bilateral striatum, left posterior cingulate cortex, right prefrontal cortex, left cerebellum and right ventroposterior lateral thalamus in the OMT group as compared with Sham. OMT showed a statistically significant negative correlation between baseline High Frequency HRV changes and rCBF changes at T2 in the left posterior insula and bilateral lentiform nucleus. The same brain regions showed a positive correlation between rCBF changes and Low Frequency HRV baseline changes at T2. These findings suggest that OMT can play a significant role in regulating brain–heart interaction mechanisms.

## Introduction

Osteopathy is a wide-spread complementary medicine that steadily increased during the last decade, especially in pain treatment^1^. Several clinical studies explored the effects of osteopathic manipulative treatment (OMT) in different pain conditions and specifically in low back pain (LBP). OMT showed significant benefit on pain intensity^2^, functional disability^3^, health-related quality of life^4^, functional recovery^3,5^ and drugs consumption^6,7^. A recent systematic review showed that the use of OMT is clinically effective in pain relief (mean difference − 12.91; 95% CI − 20.00 to − 5.82) and functional status (standardized mean difference − 0.36; 95% CI − 0.58 to − 0.14) in acute and chronic nonspecific LBP^8^. These data led to the updating of US Guidelines for OMT in LBP patients, establishing the clinically relevant effects of OMT^9^. However as suggested by the Task Force on the Low Back Pain Clinical Practice Guidelines “more research is needed to understand the mechanics of OMT and its short- and long-term effects”^9^.

Short-term neurobiological effects seem to show that OMT has a peripheral parasympathetic anti-inflammatory effect^10–19^.

Despite these clinical studies, only few papers were published investigating the effect of OMT using MRI. Recently, using Blood Oxygen Level Dependent (BOLD) fMRI, Cerritelli and colleagues showed that 4 sessions of osteopathic treatment induce a specific and distinct effect on brain activation in areas related to interoception (bilateral insula and anterior cingulate cortex) as well as in the right middle frontal gyrus and left striatum^20^.

However, there is only one study that used MRI and regional cerebral blood flow (rCBF) to investigate the post-treatment effects of OMT in asymptomatic volunteers^21^. Tamburella and colleagues showed an effect of OMT on rCBF, indicating that brain perfusion was reduced in the posterior cingulate cortex and superior parietal lobule immediately after treatment, implying a possible involvement of the central autonomic network, indicating a potential central role of autonomic nervous system (ANS). However, the authors stressed the need to investigate the effects of OMT on both the ANS and CBF. The current study is designed to meet this need by using Arterial Spin Labeling (ASL) to assess regional brain perfusion, and simultaneous measurement of cardiac and respiratory activity to derive ANS indexes such as the heart rate variability (HRV).

Although fMRI based on the BOLD contrast can detect subtle signal variations due to graded stimulation with high sensitivity^22–24^, it is unable to offer a quantitative measure of brain activity associated to a single condition. In this regard, BOLD is severely limited when it is not possible to alternate control and experimental conditions. This is particularly true for chronic pain studies, where it is not possible to switch on and off different pain levels at will as required by standard BOLD paradigms. In contrast, noninvasive measurement of regional CBF using ASL^25^, offers the opportunity to obtain a measure of a well-defined physiological variable that can quantify both baseline and task induced variation of brain activity. Importantly, CBF can be measured for each condition independently, even when they are separated by days or months with good long term reproducibility^26^, making ASL a technique naturally suitable to study chronic pain or aging^27–29^. Thus, examining changes in rCBF over time has been considered a relevant option to study pain processing^29–31^. In this study we use a combination of measures including rCBF and HRV to investigate brain and heart rate correlates of changes in baseline levels of chronic LBP (cLBP) after OMT.

A recent meta-analysis of fMRI data reported that chronic pain patients showed a major role of the following structures related to the pain matrix: bilateral precentral (BA6&BA44) and postcentral gyri (BA40), bilateral insula, left S2, left dorsal ACC (BA32), left cingulate gyrus, right medial frontal gyrus (MFG), left anterior cerebellum, bilateral basal ganglia and bilateral thalamus^32^.

According to Tanasescu and colleagues, the most frequently reported brain areas involved in chronic pain were the right posterior insula (BA13) (63% of all chronic pain research), the left BA13 and the bilateral inferior parietal lobule (S2; BA40) in 37–50% of all studies. About a third of the study reported bilateral putamen as well^32^.

Interestingly enough, some of these brain areas, such as bilateral midcingulate cortex, left posterior and right anterior insula and left amygdala, are recognized as core regions of the central control of autonomic system^33^. Indeed, Beissner et al. synthetized, in a seminal paper, the features of the so called central autonomic network (CAN), which controls the sympathetic and parasympathetic divisions that have largely divergent specific regulatory networks, differentially involved in affective, cognitive, and somatosensory–motor tasks^33^.

We hypothesized that the OMT, as compared to the control condition, would induce a decrease in rCBF in a widespread network of brain regions, including (but not exclusively) those of the pain matrix and an increase in parasympathetic activity as measured by HRV.

## Results

### Description of the sample at the baseline

Thirty-two right-handed patients were randomised and divided into the study (N = 16) and control group (N = 16). Two patients (1 in the study group and 1 in the control group) dropped out during the study, leading to a final sample size of 30 patients (Figure S1). At the baseline, there were no statistically significant differences between groups in terms of age, gender, BMI and clinical characteristics of pain in (Table 1). Only the 10% of the patients correctly guess the group allocation (OMT: 2/15; Sham: 3/15; X^2^ = 0.24, p = 0.62).

**Table 1:** Clinical characteristics of study population.

	Study group (OMT)	Control group (Sham)	p < |t|
Age	41.8 ± 6.6	42.7 ± 8.0	0.73
Male	9 (60)	11 (73.3)	0.70*
BMI	24.1 ± 3.5	25.5 ± 2.4	0.19
LBP duration (m)	15.1 ± 9.2	14.1 ± 6.7	0.72
**General scores**			
STAI-Y1	42.4 ± 3.4	42.7 ± 2.9	0.85
STAI-Y2	41.3 ± 3.0	41.1 ± 3.7	0.87
TEMPS-A	8.8 ± 3.4	9.27 ± 4.1	0.48
**Pain scores**			
VAS—T0	63.1 ± 21.4	57.5 ± 17.3	0.10
VAS—T1	31.3 ± 21.7	47.5 ± 15.4	< 0.001
VAS—T2	18.3 ± 20.5	53.7 ± 23.6	< 0.001
Oswestry—T0	24.9 ± 3.3	26.0 ± 5.2	0.51
Oswestry—T1	16.4 ± 2.6	25.2 ± 4.1	< 0.01
Oswestry—T2	10.8 ± 2.1	24.5 ± 3.7	< 0.001
Roland-Morris—T0	15.5 ± 4.0	15.3 ± 4.9	0.90
Roland-Morris—T1	11.4 ± 3.4	15.0 ± 3.8	< 0.01
Roland-Morris—T2	8.7 ± 2.6	14.9 ± 4.6	< 0.001

Numbers in table are mean ± SD or *N(%). P values from Student *t* test and *Chi square. *BMI* body mass index, *LBP* low back pain, *VAS* visual analogue scale.

At the end of the study period, the pain scales showed significant differences between the two groups (Table 1).

Table 2 reports rCBF values among the different groups and time points.

**Table 2:** rCBF values.

ROI	OMT			SHAM		
	T0	T1	T2	T0	T1	T2
Left posterior insula	52 ± 3	52 ± 2	47 ± 5	51 ± 3	52 ± 4	50 ± 2
Left anterior cingulate cortex	52 ± 3	51 ± 2	45 ± 5	50 ± 2	51 ± 5	49 ± 3
Left posterior cingulate cortex	46 ± 2	46 ± 2	51 ± 5	46 ± 2	47 ± 2	46 ± 3
Left lentiform nucleus	49 ± 2	49 ± 1	51 ± 3	50 ± 2	49 ± 3	47 ± 2
Left middle frontal lobe	49 ± 4	49 ± 3	46 ± 5	48 ± 3	47 ± 4	47 ± 4
Left cuneus	51 ± 3	48 ± 3	46 ± 4	49 ± 3	48 ± 4	48 ± 2
Left superior parietal lobe	50 ± 4	49 ± 4	53 ± 5	49 ± 4	48 ± 7	46 ± 4
Right lentiform nucleus	48 ± 2	48 ± 2	49 ± 4	48 ± 3	48 ± 2	46 ± 1
Left thalamus	49 ± 2	49 ± 3	46 ± 2	49 ± 3	49 ± 2	51 ± 2
Left cerebellum (crus 1)	44 ± 5	43 ± 4	47 ± 5	42 ± 3	42 ± 3	40 ± 1
Right anterior cingulate cortex	52 ± 3	51 ± 3	54 ± 2	54 ± 3	54 ± 3	50 ± 3
Right ventral anterior insula	51 ± 2	50 ± 2	53 ± 3	53 ± 2	54 ± 5	50 ± 3
Right dorsal anterior insula	51 ± 4	50 ± 3	53 ± 4	53 ± 4	55 ± 5	52 ± 4
Right mid orbital gyrus	51 ± 3	50 ± 2	53 ± 2	52 ± 3	53 ± 3	50 ± 4
Right orbito frontal cortex	41 ± 1	42 ± 1	43 ± 3	42 ± 2	43 ± 3	41 ± 2
Right ventro postero lateral thalamus	46 ± 2	46 ± 2	48 ± 2	48 ± 2	48 ± 2	46 ± 2

Regional CBF values on region of interests (ROI) in both study groups at different time points. Values are expressed in ml/100 g/min. *T0* baseline, *T1 *immediately after the first session, *T2 *at the end of the study period.

### The effect of osteopathic treatment on CBF

The control contrast T0_OMT vs T0_SHAM revealed no significant differences between the two groups for rCBF values at baseline.

**Figure 1:**
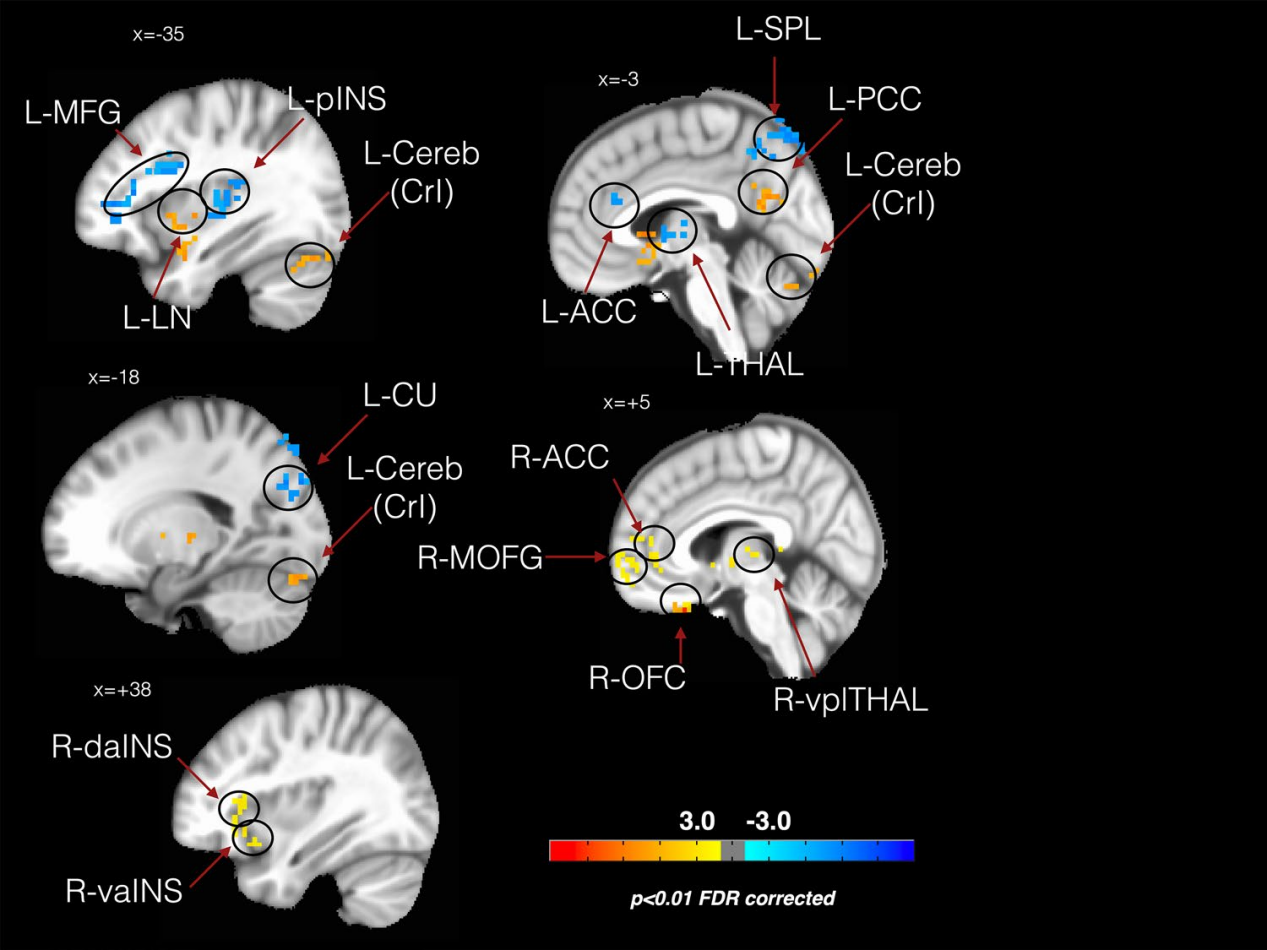
The effects of osteopathic treatment on regional cerebral flow. The figure shows CBF changes baseline-controlled group differences between treatment and sham group at T2 (referring to the contrast described in the text as (T2_OMT–T0_OMT) vs (T2_SHAM–T0_SHAM)—*p* < 0.01, false discovery rate (FDR) corrected).* L-MFG* left middle frontal gyrus, *L-pINS* left posterior insula, *L-LN* left lentiform nucleus, *L-Cereb*(*CrI*) left cerebellum (Crus I), *L-ACC* left anterior cingulate cortex, *L-SPL* left superior parietal lobe, *L-PCC* left posterior cingulate cortex, *L-THAL* left thalamus, *L-CU* left cuneus, *R-ACC* right anterior cingulate cortex, *R-MOFG* right mid orbitofrontal gyrus, *R-OFC* right orbito frontal cortex, *R-vplTHAL* right ventroposterior lateral thalamus, *R-daINS* right dorsal anterior insula, *R-vaINS* right ventral anterior insula.

The whole brain analysis (Fig. 1) showed differences between groups in CBF values on specific regions only for the following contrasts (T2_OMT–T0_OMT) vs (T2_SHAM–T0_SHAM) and (T2_OMT–T1_OMT) vs (T2_SHAM–T1_SHAM): the left posterior insula (L-pINS), right ventral anterior insula (R-vaINS), right dorsal anterior insula (R-daINS) left anterior cingulate cortex (L-ACC), left superior parietal lobe (L-SPL) and the left middle frontal lobe (L-MFL, a subregion of the left frontal cortex), the left and right striatum, specifically the lentiform nuclei (L-LN, R-LN), the left posterior cingulate cortex (L-PCC), the left cuneus (L-CU), left thalamus (L-THAL), right ventroposterior lateral thalamic nucleus (R-vplTHAL), right orbito-frontal cortex (R-OFC), right anterior cingulate cortex (R-ACC), right mid orbital frontal gyrus (R-MOFG) and the left cerebellum crus 1 (L-Cereb(CrI)).

**Figure 2:**
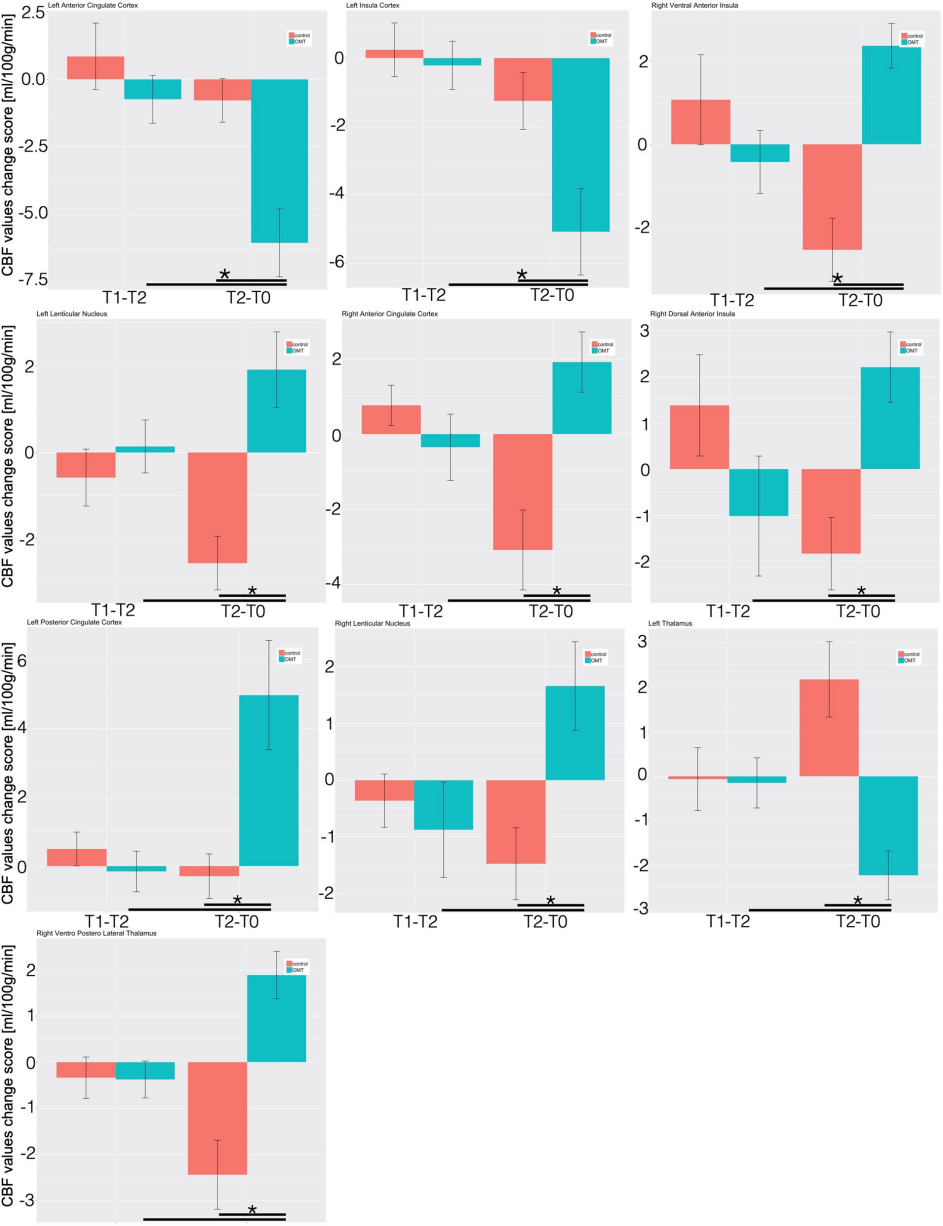
Longitudinal CBF delta changes. The figure shows the longitudinal CBF mean change within the regions of interest for the two groups. *Statistically significant differences (p < 0.05) between groups.

Considering the region of interest (ROI) analysis, the mixed effect regression (MER) analysis showed that, as compared with control group, patients that received OMT demonstrated decreased regional CBF in the L-pINS, L-ACC, L-MFL, L-THAL and the L-CU (Fig. 2 and Table S1).

Conversely, rCBF was increased in the L-LN, R-LN, L-PCC, L-SPL, R-vaINS, R-daINS, R-vplTHAL, R-OFC, R-ACC, R-MOFG and L-Cereb (CrI) in OMT patients as compared with controls (Table S1, Fig. 2 and Figure S2).

Post-hoc analysis revealed that these changes were statistically significant only at T2 and not immediately after the first treatment session.

Table S2 showed post-hoc effect size computation with a large effect size for the majority of the ROI. Besides, post-hoc calculation of study power demonstrated an adequate power for almost all the ROI included.

### Relationships between rCBF and self-reported pain

Considering regions in which CBF changes due to treatment were significantly different between groups, 6 ROIs showed a significant correlation of CBF variation with variation of VAS pain score (both evaluated for T2–T0) (Table 3). This significance was demonstrated to be only present in the OMT group and not in the control group.

**Table 3:** Correlation values between baseline change response of rCBF and VAS pain score at T2.

	OMT	p values	SHAM	p values
Left posterior insula	0.55	**0.03**	− 0.07	0.79
Left anterior cingulate cortex	0.51	**0.05**	0.14	0.62
Left posterior cingulate cortex	− 0.52	**0.04**	0.05	0.84
Left lentiform nucleus	0.35	0.20	0.16	0.58
Left middle frontal lobe	0.51	**0.05**	− 0.36	0.20
Left cuneus	− 0.04	0.87	0.05	0.84
Left superior parietal lobe	− 0.06	0.83	0.33	0.25
Right lentiform nucleus	0.53	**0.04**	− 0.27	0.35
Left thalamus	− 0.62	**0.01**	− 0.07	0.80
Left cerebellum (crus I)	0.19	0.50	0.30	0.28
Right anterior cingulate cortex	− 0.40	0.13	− 0.25	0.37
Right dorsal anterior insula	− 0.14	0.61	− 0.04	0.88
Right ventral anterior insula	− 0.08	0.75	0.23	0.40
Right mid orbital gyrus	0.04	0.89	− 0.27	0.32
Right orbitofrontal cortex	− 0.30	0.28	0.17	0.53
Right ventro postero lateral thalamus	0.27	0.33	− 0.02	0.94

Bold values showed statistically significant changes.

**Figure 3:**
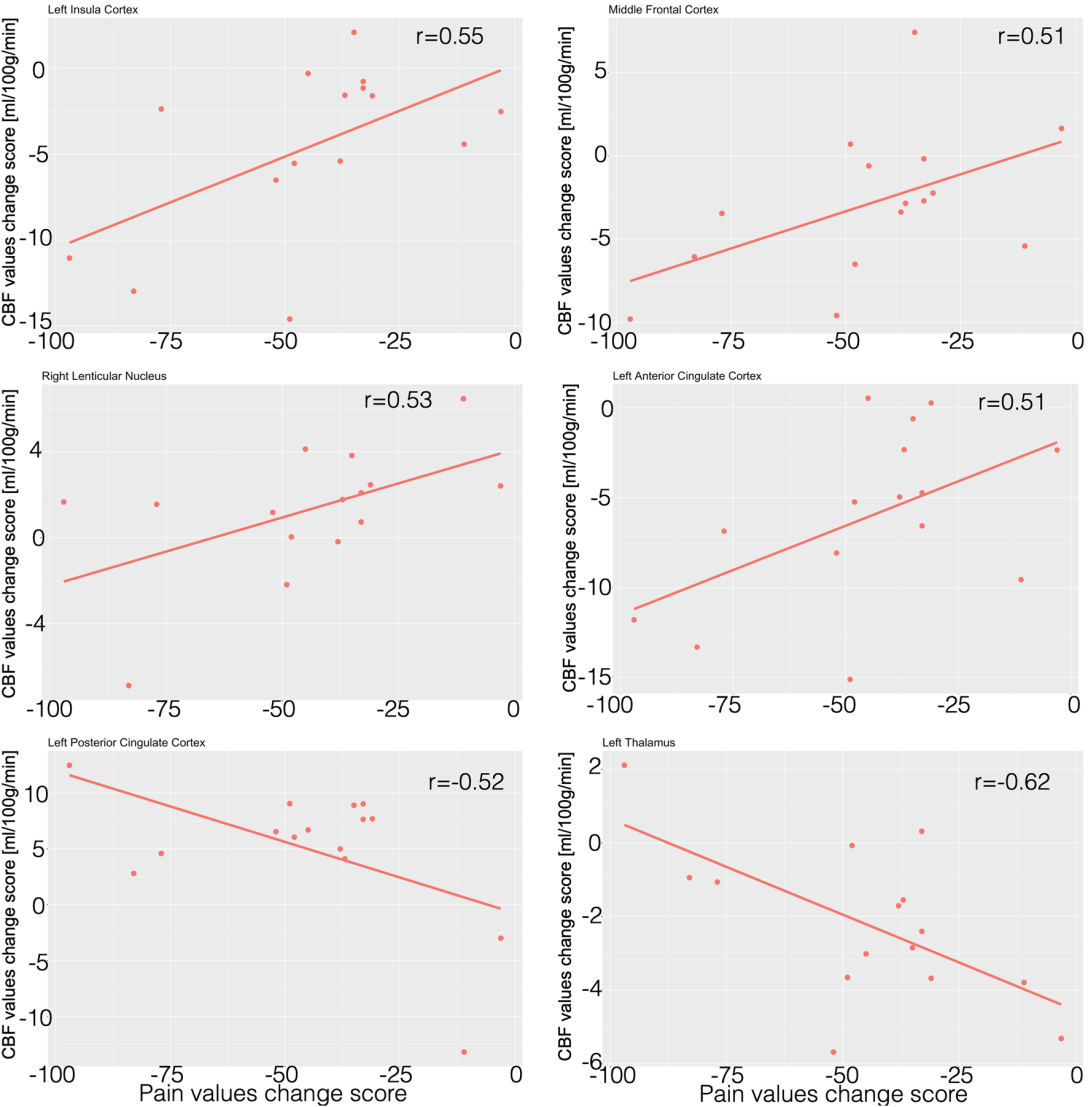
Correlation between rCBF and pain. The figure shows the significant positive and negative correlations between regional cerebral blood flow mean changes within ROI and pain intensity changes in the treatment group.

Specifically, baseline CBF changes at T2 in the OMT group were significantly correlated with the corresponding changes in VAS scores in the L-pINS (r = 0.55; p = 0.03), L-ACC (r = 0.51; p = 0.04), L-MFL (r = 0.50; p = 0.04), R-LN (r = 0.53; p = 0.04), L-PCC (r = − 0.52; p = 0.04) and L-thalamus (r = − 0.62, p = 0.01) (Fig. 3).

No correlation was demonstrated to be statistically significant at T1, i.e. immediately after the first session, for both groups.

### HRV results

**Figure 4:**
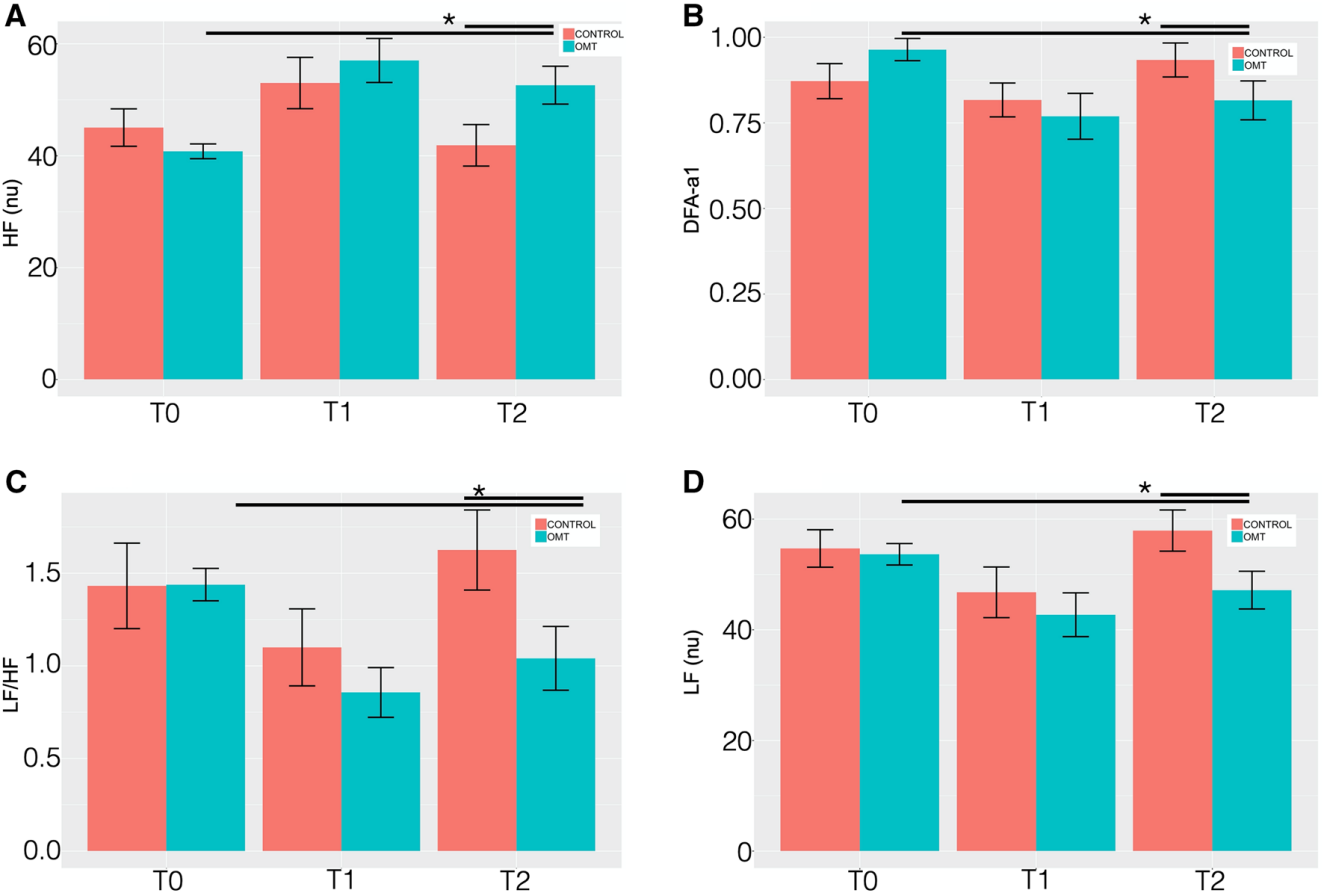
Heart rate variability changes between the study and control group. Heart rate variability (HRV) findings for: (**A**) high frequency (HF) normalised units (nu); (**B**) detrended fluctuation scaling exponent (DFAα1); (**C**) low frequency/high frequency ratio (LF/HF); (**D**) low frequency (LF—nu). Data presented are means ± standard deviation (SD). *Statistically significant differences (p < 0.05) in OMT group compared to sham and control groups.

MER showed a statistically significant difference between groups on the nuHF (*p* < 0.001) (Fig. 4). Tukey post-hoc analysis revealed that OMT group significantly increased nuHF-values compared to sham at T2 (*p* < 0.01) but not at T1.

Similarly, statistically significant differences were revealed for nuLF (p < 0.01), LH/HF ratio (p < 0.01) and DFA-a1 (p < 0.05). Tukey post-hoc analysis demonstrated that in all the HRV parameters the OMT group had a significant effect compared to sham at T2 but not at T1.

In addition, other time domain parameters (Table S3) showed similar results, including heart rate (p < 0.05), RMSSD (p < 0.05), NN50 (p < 0.01), pNN50 (p < 0.001). Sample entropy did not show any statistically significant difference (p = 0.19).

### Relationships between rCBF and HRV parameters and pain

**Figure 5:**
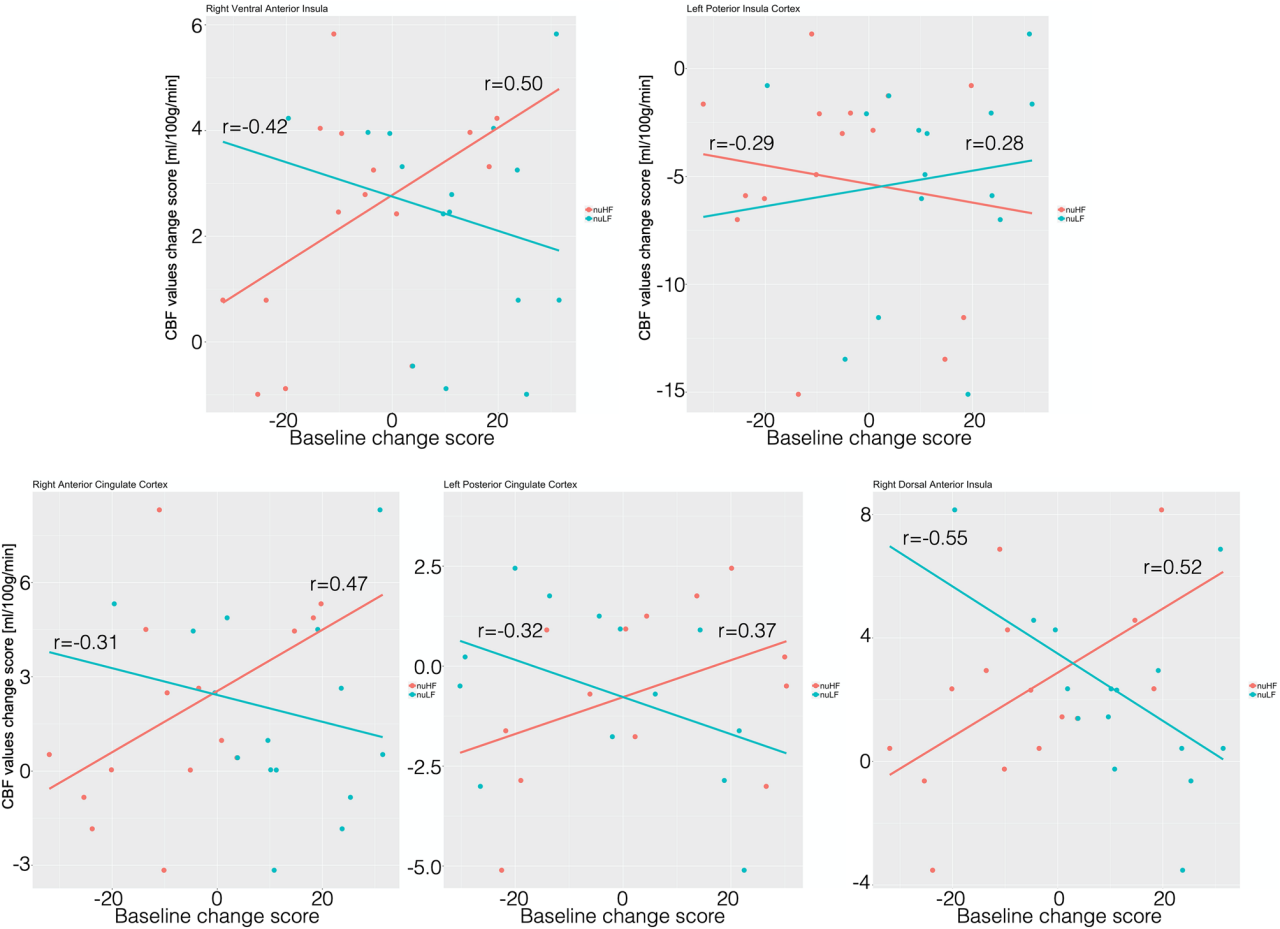
Correlation between heart rate variability changes and rCBF in the study group. The figure shows the significant positive and negative correlations between regional cerebral blood flow mean changes within ROI and nuHF (red line) and nuLF (cyan line) changes in the treatment group.

Only in the OMT group, a statistically significant negative correlation (Fig. 5) was found between rCBF changes and baseline nuHF changes at T2 in L-pINS (r = − 0.29; p = 0.05), whereas positive correlations were shown for L-PCC (r = 0.37, p = 0.03), R-vaINS (r = 0.50, p = 0.01), R-daINS (r = 0.52, p = 0.01) and R-ACC (r = 0.47, p = 0.02).

Furthermore, the same brain regions showed opposite correlations at T2 between rCBF changes and nuLF changes: L-pINS (r = − 0.28; p = 0.05), L-PCC (r = − 0.32, p = 0.05), R-vaINS (r = − 0.42, p = 0.03), R-daINS (r = − 0.55, p = 0.01) and R-ACC (r = − 0.31, p = 0.05).

Also, data showed a positive correlation between nuHF and pain (r = 0.27; p = 0.002) in the OMT group but not in the control sample (r = − 0.17; p = 0.08).

Again, no statistically significant correlations were depicted at T1 between all parameters above-mentioned.

## Discussion

Results from this trial showed that OMT has a two-fold effect: central, that is a change in the brain by modifying the CBF in some but not all the areas related to the pain matrix, and peripheral, that is an autonomic effect as measured by a change in HRV, specifically on HF and DFA-a1.

To our best knowledge, this is the first experimental evidence of such combined modifications, providing suggestions that a mutual interconnection response might mainly mediate this brain-and-heart effect due to the administration of osteopathic treatment.

After the OMT sessions there was a significant CBF decrease in some brain areas related to pain, that is L-pINS, L-ACC and L-thalamus, an increase of CBF values in the L-PCC, lentiform nuclei, R-vaINS, R-daINS and R-vplTHAL (Figs. 2, 3 and Table 2). In addition, the CBF modification in 6 pain-related areas showed significant correlations with reduction of baseline VAS pain in the OMT group but not in the controls. Specifically, positive correlations were found on L-pINS, L-ACC, L-MFL, right lentiform nucleus, whereas a negative correlation was detected for the L-PCC and L-thalamus.

These findings expand the results reported from previous fMRI studies in the same group of cLBP patients^20^ and in asymptomatic healthy subjects^21,34–36^. Interestingly, Tamburella and colleagues reported that a single OMT session produced an immediate blood flow changes in L-PCC and L-SPL^21^. The present study extends this previous work by examining effects in chronic LBP patients, over an extended period and combining HRV measurements. That is, while previous research has used CBF measurements immediately after a single OMT session and after three days, here we report not only that larger CBF changes are observed in response to a longer osteopathic treatment period (4 sessions over a 1-month treatment period) but that these effects are linked to a change in autonomic response. We interpret the accompanying decrease of CBF in pain-related areas as reflecting the positive influence of osteopathic treatment on heart rate variability and pain perception on LBP patients’ physiological state, hypothesizing a potential OMT effect on central mechanisms of endogenous pain modulation.

Further support of our hypothesis that OMT might act on CBF through the ANS by reducing the inflammatory milieu of the tissue comes from the analysis of the CBF values in combination with autonomic reactions.

Our findings are in line with previous studies demonstrating an increase in CBF on areas related to chronic pain. Indeed, the increase of CBF in pain-related areas was also observed in neuralgia pain patients and positively correlated to pain intensity^37^. The insula, S1, and thalamus were previously linked to both acute and chronic pain^38,39^, and it was reported that these regions encode discriminative sensory components of acute pain in terms of both intensity and somatotopy^40–42^. Additionally, INS and S1 consistently show increased activity in chronic pain^43,44^. A 2016 meta-analysis demonstrated that in chronic pain studies the following areas were most frequently reported: bilateral INS—in particular the right posterior insula and the left insula, left inferior parietal lobule, left dorsal ACC (BA32), bilateral precentral (BA6&BA44) and postcentral gyri (BA40), right medial frontal gyrus, bilateral basal ganglia, i.e. putamen, bilateral thalamus and left anterior cerebellum^32^. Thus, CBF increases in several pain conditions, specifically in the pain regions. Our data showed that the use of 4 sessions of osteopathic treatment produced significant variations of baseline CBF values in some but not all the areas related to pain. This result might mean distinct regulation of the blood flow with a specificity of response, that is a decrease in L-pINS, L-ACC and left thalamus and an increase in L-PCC, right anterior insula, bilateral basal ganglia, right thalamus.

Nevertheless, these variations might be linked to significant changes in the control of the central autonomic network (CAN). Beissner and colleagues in a 2013 metanalysis focusing on CAN suggested that the prefrontal, anterior, and midcingulate, right ventral anterior insular and left posterior insular cortices are involved with sympathetic activity whereas the PCC, lateral temporal cortices, bilateral dorsal aINS with parasympathetic responses^33^. These brain areas appear to be distinctively associated with cognitive output (ACC, INS, SPL) or to somatosensory output (PCC) of CAN. These findings were recently confirmed and extended by Valenza et al., who identified additional regions such as the precuneus, angular gyrus, and cerebellum^45^.

Therefore, we might argue that the central effect of OMT—which elicited a change in brain perfusions within specific areas also related to CAN, specifically bilateral ACC, R-vaINS, R-daINS, L-pINS, L-PCC—can be linked to the regulation of CAN on both sympathetic and parasympathetic components. Beissner et al.’s proposal on the distinction between sympathetic and parasympathetic might explain the different behaviours we found on CBF reactions in the current study^33^. However, we need to consider that Beissner and colleagues^33^ as well as Valenza and colleagues^45^ summarized studies mainly based on healthy subjects, with and without CBF data, in rest and task-based paradigms. Interestingly, Chouchou et al., recently showed that stimulation of the anterior insula mainly induced a cardiovagal-mediated decrease in the heart rate, whereas stimulation of the posterior insula mostly produced a sympathetically mediated increase^46^. This might also be consistent with our results where the OMT increased the perfusion of the right anterior insula, reduced the perfusion of the L-pINS and increased nuHF, reflecting a decrease in heart rate.

Thayer and colleagues originally proposed the association between CAN and HRV within the Neurovisceral Integration Model (NVI), a model where the variability of heart rate, as measured by HRV, is considered a valuable proxy for the brain state^47^. This model was further developed recently by Smith and colleagues who better explored and contextualized the NVI model proposing eight neurofunctional hierarchical levels of control over the vagus nerve (see Smith et al. for details^48^).

In particular, relevant for this discussion about the role of vagus nerve as parasympathetic output and central autonomic control, is the level 6, which includes INS and ACC, that regulates processes incorporating perceptual representations of one’s current bodily state, and level 7, which includes the PCC, that further regulates these processes embracing conceptual interpretations of the meaning of both exteroceptive and interoceptive sensory input. In sum, these higher-order brain areas hierarchically regulate the neurovisceral system by, among others, controlling the cardiac autonomic output. Therefore, the fact that we observed cardiac autonomic effects on chronic pain population and that these sequelae are associated to central patterns generates discussion on (i) changes in the cross-organs relationship, more specifically brain and heart, after OMT, (ii) the relationship between brain, heart and peripheral tissue.

Interestingly, examples of coordinated cross-organ reactions include the impact of cardiac functions on cerebral activity^49,50^. Thus, modulating the cardiac output by a parasympathetic effect produces a direct effect on cerebral activity. Our data showed that a sympathetic response is associated with a decrease of CBF in some brain areas related to the pain matrix. This means that a relative reduction of blood flow might be arguably produced based on a sympathetic response. This might be conceptually understood as pain patients express higher baseline CBF values in these regions compared to asymptomatic-healthy subjects.

Clinical and laboratory research demonstrated the role of sympathetic branch in preventing increase of CBF^51–54^, acting as a neurogenic modulator when hemodynamic changes challenge cerebral activity. Therefore, applying a treatment, which produces a reduction of pain, might justify lower perfusion of these areas and thus a relative decrease of blood flow^51^. On the contrary, exploring the cardiac data, the findings of the present study are consistent with previous research on adults, which concluded that osteopathic approach produces an increase in parasympathetic tone leading to a trophotropic effect^12^. Thus, a double effect seems to be produced where a prevailing sympathetic response is observed centrally, whereas a major parasympathetic effect is showed peripherally.

To further explore this hypothesis, it has been argued that the osteopathic approach might act through an anti-inflammatory and hyperparasympathetic effect mediated by interoception^13^. While we did not include a measure of pro-inflammatory levels in this study, it has previously been reported that in a sample of LBP patients the use of OMT can reduce cytokines levels^16^ and this reduction is associated to a reduction of pain perception^15^. Our sample showed a significant reduction of pain perception and better overall clinical conditions, possibly related to a reduction of pro-inflammatory state in the tissue leading to a change in the neurogenic neuroinflammation^55^. The ANS, CAN and interoception mediate all these processes. Modifying the metabolic state of the tissue, therefore, produces a proxy for unmyelinated c-fibers that in turn, through the spinothalamic tract, results into the activation of the insular cortex, specifically the posterior lobe of the left insula^56–59^.

Interestingly, our data showed that the left posterior INS is a specific target of the osteopathic effect. Also, the other areas involved after OMT are consistent with the hypothesis of the awareness model^58^, where the insula receives input from other areas of the brain including hedonic areas (PCC, ACC). Therefore, we might argue that the use of osteopathic treatment might produce a significant change in the cerebral activity, specifically on key areas related to CAN and thus to interoception and interoceptive control.

The present study only examined the immediate and 1-month sustained effect of OMT on chronic pain patients. Future work is needed to determine whether there are longer-term, clinically and brain significant, benefits of this type of manual intervention and if so, what dose, in terms of frequency and duration, is required. Systematic evaluation of the effects of OMT on chronic pain patients by Cerritelli and colleagues have determined that eight treatment sessions over a 6 month period are sufficient to decrease pain, disability and migraine attacks per month^60^. Therefore, comparing immediate effects with long-term outcomes would shed light on the brain changes that might occur. Furthermore, although the post-hoc analysis showed an adequate power for the majority of the ROIs, future CBF studies might benefit from an a priori power calculation.

In conclusion, osteopathic interventions can have some clinical benefits, but a greater understanding of the neurobiological mechanisms is needed to optimize pain protocols and ameliorate the long-term negative consequences of pain. The present study supports the hypothesis that OMT can induce CBF changes by acting through autonomic responses, offering insight into the development and optimization of novel pain care strategies.

## Methods

The study was designed as randomised placebo-controlled to explore possible mechanisms to explain the effect of osteopathic manipulative treatment compared to sham treatment on cerebral blood flow in a sample of cLBP patients.

### Subjects

Adult patients with chronic Low Back Pain were recruited from a rehabilitation centre of the university of Chieti (CUMFER). Inclusion criteria included any chronic (> 3 months) pain or discomfort localised below the costal margin and above the inferior gluteal folds, with or without referred leg pain^61^. Exclusion criteria were: clinical sign of neurological damage with sensorimotor impairments (i.e. radicular syndrome, paresis or tingling in limbs); suspected or confirmed spinal pathology (e.g. tumour, infection, fracture or inflammatory disease); history of spinal surgery (e.g. decompensation or stiffening); whiplash incidence within the last 12 months; cervical pain that reduces active movement to less than 30° rotation to each side; known vestibular pathologies; major surgery scheduled during treatment; physiotherapy during the last 12 weeks; inability to follow the procedures of the study: e.g. due to language problems, psychological/psychiatric disorders, dementia of the participant; parallel participation in another study. At enrolment, eligible patients were assessed by a senior MD to confirm the diagnosis and exclude psychiatric disease and/or any other exclusion criteria.

Eligible patients were randomly divided into two groups using a 1:1 ratio and assigned to the study (OMT) group or the sham group. Block randomization was applied according to a computer-generated randomization list using a block size of 10. All patients were not aware of any step of the study design as well as outcomes or group allocation. The randomization list was stored in a dedicated and protected web-based space and an information technology consultant was in charge for the entire process.

Research staff were unaware of the study design and outcomes. Moreover, they were blinded to patients’ allocation, since all patients were touched by the practitioner. Only the osteopath was aware of the patients’ allocation. Moreover, the practitioner who performed OMT had no role in patient care decisions. Researcher using the MRI and dealing with MRI data were unaware of patients’ allocation.

### Ethics and reporting

The study was approved by the local ethics committee (University of Chieti-Pescara number: 7/09-04-15) and all subjects provided informed written consent, in line with the Declaration of Helsinki. The current protocol was registered on clinicaltrial.gov (ID: NCT02464475) on 08/06/2015. All methods were carried out in accordance with relevant guidelines (CONSORT, TIDIER and SPIRIT) and regulations.

### Prescan assessment

Before the MRI session, patients were asked to complete paper-based questionnaires. A socio-demographic questionnaire was used to collect baseline data.

The Edinburgh Handedness Inventory was utilized to investigate the hand dominance^62^. The State-Trait Anxiety Inventory (STAI-Y1 and Y2) was used to test trait anxiety^63^.

Furthermore, the TEMPS-A (Temperament Evaluation of the Memphis, Pisa, Paris and San Diego—Autoquestionnaire) was administered to measure the affective temperament that defines the bipolar spectrum, with depressive (D), cyclothymic (C), hyperthymic (H), irritable (I), and anxious (A) subscales^64–66^.

Several tools were specifically used to assess pain perception in patients: Visual Analogue Scale (VAS), the Roland–Morris Disability Questionnaire and the Oswestry Low Back Disability Questionnaire (OSW) for the physical disability due to low back pain^67,68^. Questionnaires were administered at enrolment, at each session and at the end of the study period.

### Experimental procedure

Patients eligible for the study were divided and randomised in an OMT and Sham group. Patients allocated to the OMT group received 4 osteopathic sessions (approximately 30 min each). Osteopathy is a drug-free manual medicine, where osteopathic practitioners use manual techniques to diagnose and treat somatic (body framework) dysfunction (ICD-10 code: M99.0–99.9)^69^. Osteopathy is based on the structural assessment to diagnose somatic dysfunctions and a series of manipulative techniques for the treatment^70^.

In the present research the treatment was administered by a licensed and registered osteopath. Among the series of techniques, the techniques used in the current study were balanced-ligamentous, balanced-membranous and fluidic techniques, in line with the principles and procedures available in the current osteopathic literature. All treatment sessions took place in the CUMFER.

The sham group received an osteopathic-like manual assessment and treatment, that means the practitioner applies a manual contact without using any type of specific osteopathic technique or procedure. After the evaluation, the operator asked the patient to lay down on the plinth and gently placed the hands on a pre-defined set of bodily parts without applying any type of technique but just using a gentle static or dynamic touch. The parts identified in the protocol were: low back, sacrum, pelvis, diaphragm, upper thorax, cervical spine and cranium. The sequence to apply during the session was decided by the operator before the session. This was planned to prevent any possible chance from the patient to guess the group allocation. The sessions lasted 30 min, as for the OMT, took place in the same location/room and were administered by the same practitioner. Besides, the operator was instructed to maintain and establish the same type of patient-doctor relationship. This procedure was used to avoid any possible contamination and to prevent allocation bias. Patients assigned to the sham group received osteopathic treatment following completion of the trial.

During the study period, all patients were asked to avoid drug consumption.

As for a previous BOLD study^20^, the 3 time points were established (Supplementary material—Figure S1):

1. Baseline (T0): before the treatment
2. Immediate response (T1): Immediately after the first manual session
3. Sustained response (T2): at the end of the study period (after a month), which included 4 treatment sessions

After the clinical evaluation (enrolment) and at T2, patients were asked to fill in the paper-based questionnaires.

### Arterial spin labeling data acquisition

MRI was performed using a Philips Achieva 3 T scanner (Philips Medical Systems, Best, Netherlands). A whole-body radiofrequency coil for signal excitation and an 8-channel phased-array head coil for signal reception were used. A high-resolution structural volume was acquired using a 3D fast field echo T1-weighted sequence (sagittal, matrix 256 × 256, FOV = 256 mm, slice thickness = 1 mm, no gap, in-plane voxel size = 1 × 1 mm, flip angle = 12°, TR = 9.7 ms and TE = 4 ms). Then, Blood Oxygen Level Dependent (BOLD) fMRI data were acquired. Perfusion imaging was performed using a pseudo-continuous ASL (pCASL) sequence^71^ with the labeling parameters optimized according to a recent white paper^72^: postlabel delay 1900 ms, label duration 1750 ms. Background suppression pulses were used at 2110 ms and 3260 ms after start of labeling. Other imaging parameters were TR/TE = 4269/10 ms, SENSE factor 2.3, matrix 64 × 64, voxel size 3.6 mm × 3.6 mm × 5 mm, 19 slices acquired in ascending order, 60 dynamics. The labelling plane was positioned 85 mm below the AC-PC line^72^. An equilibrium magnetization image (M0) with a long TR (10,000 ms) was also acquired for calibration purposes using readout parameters identical to the pCASL sequence. The total duration of the pCASL measurement was about 5 min.

The MRI session also included the acquisition of BOLD data, part of which were recently published^20^. Importantly, we carefully controlled potential confounding factors requiring the subjects to refrain from caffeine, nicotine or alcohol consumption for at least 6 h before the MRI exam. In addition, the period within the day where the acquisition was performed, and the time spent in the scanner before the ASL measurement was the same for each subject and session to exclude effects due to circadian cycle, fatigue and menstrual cycle.

During all MRI measurements, respiratory and cardiac physiological activity was recorded using the built-in scanner devices. Specifically, respiratory cycle was recorded with a pneumatic belt strapped around the upper abdomen, whereas the cardiac cycle was registered with a pulse oximeter placed on a finger of the right hand. Both signals were acquired during the pCASL sequence and sampled at 100 Hz, allowing a reliable assessment of HRV^73^.

In addition, subjects were instructed to stay awake with their eyes closed and minds clear. Maximum care was taken to avoid situations that may trigger abnormal pain. Subjects laid in a supine position on the scanner table with head immobilized by foam padding.

### ASL data preprocessing

Analysis of pCASL data was carried out using AFNI. Preprocessing was performed on the tag and control ASL images separately^74^. First, motion correction was performed to realign all tag/control time frames to a reference image represented by the first tag/control volume. Second, an additional coregistration matrix between these two reference images was determined to account for possible head motion between the two acquisitions, using a mutual information-based approach^75^. These preprocessed control/tag timeseries were coregistered to the M0 image that was also used to determine a coregistration matrix between perfusion images and the structural scan. To take into account intrasubject alignment across the three sessions, the structural scan at T0 was used as the reference image for all coregistrations. Finally, individual subject data were normalized to the MNI space using linear and nonlinear transformations (AFNI script “@SSwarper”).

Voxel-wise quantitative CBF maps were derived using a single compartment model^72,76^:

$$ CBF = \frac{{6000\lambda (SI_{C} - SI_{T} )e^{{\frac{PLD}{{T_{1A} }}}} }}{{2\alpha \alpha_{INV} T_{1A} M_{0} \left( {1 - e^{{ - \frac{\tau }{{T_{1A} }}}} } \right)}}\;({\text{ml/}}100\;{\text{g/min}}) $$

Here, SIC and SIT are the time averaged signals of control and tag images respectively, PLD is the post label delay (1900–2800 ms, depending on the slice), T1A is the longitudinal relaxation time of arterial blood (1650 ms at 3 T), α = 0.85 is the labeling efficiency^72^, αinv = 0.83 is a correction factor for the background suppression^77^, M0 is the equilibrium magnetization signal, τ is the label duration (1750 ms) and λ is the blood–brain partition coefficient (0.9 ml/g).

The calculated CBF maps were then normalized to the MNI space using the previously determined spatial transformations.

The following contrasts between voxel-wise CBF maps were considered: (a) (T1_OMT–T0_OMT) vs (T1_SHAM–T0_SHAM) describing the post first treatment vs. baseline, (b) (T2_OMT–T0_OMT) vs (T2_SHAM–T0_SHAM) showing the end of study period vs. baseline, (c) (T2_OMT–T1_OMT) vs (T2_SHAM–T1_SHAM) defining the end of study period vs. post first treatment. These contrasts represent treatment’s effects controlled by baseline, i.e. T0, scans, and delayed effect, respectively. The additional contrast T0_OMT vs T0_SHAM was performed to control for potential differences between groups at baseline. The group statistical maps obtained from these contrasts were thresholded at *p* < 0.01 corrected for multiple comparisons using False Discovery Rate (FDR).

### HRV data

Standard 5 min recording (corresponding to the pCASL acquisition) was used to derive HRV data. Inter-beat intervals were extracted from the pulse oximeter. Using customized software (Kubios automatic artefact correction algorithm function based on nonlinear predictive interpolation), outliers, i.e. artefacts and ectopic peaks, were identified and removed from the data. Specifically, to account for non-experimental movements that might produce extreme values, individual participant’s datapoints that were more than three S.D.s above or below the whole sample mean were identified. Such datapoints were determined to be additional artefacts and replaced by the mean of that participant’s non-artifactual epochs. The artifact percentage accepted for each individual was set at 2%. Two participants (1 in the OMT group and 1 in the SHAM group) were identified as having artifactual epochs, corresponding to 6.25% of period datapoints across participants. Intervals were then imported in Kubios software (http://kubios.uef.fi) to compute HRV parameters. Standard 5-min recordings were considered for RR series. There were no significant differences in the percentage of artifactual epochs identified between the two groups.

HRV analysis method, based on processing recorded RR intervals, was divided into linear analysis (time and frequency domain) and nonlinear analysis^78^. The correction method was automatic as mentioned above, with an acceptance threshold of 2%. The signal type was PPG. Regarding the QRS detection options, R-wave polarity and prior guess for RR interval were set as automatic. The RR time series interpolation rate was set at 4 Hz and the detrending method was the Smoothness priors, choosing a smoothing parameter equal to 500. The general analysis settings included: (1) Standard analysis (all time-domain, frequency-domain and nonlinear analysis for selected stationary samples), and (2) Time-varying analysis. For time-domain analysis methods, the window width of the moving average filter was set at 5 beats. Also, the threshold used in the computation of NNxx and pNNxx parameters was set at 50 ms, thus NN50 and pNN50). From power spectra (Fast Fourier transformation -FFT- using Blackman Harris window) of equidistant linear interpolated (4 Hz) tachograms (resampled to 2 Hz), the following frequency domain standard HRV indices were used for linear analysis: nuHF, from 0.15 to 0.4 Hz, i.e. signal of parasympathetic heart rate modulation^79,80^; nuLF, from 0.04 to 0.15 Hz, i.e. predictor of sympathetic modulation^81^; LF/HF ratio as being demonstrated a valid marker of ANS activity^82^. In addition, the spectrum estimation option was 300 points/Hz, the FFT spectrum using Welch’s periodogram method has a window width of 300 s and a window overlap of 50%. The AR spectrum used an AR model order of 16. The nonlinear analysis options included embedding dimension at 2 beats with a tolerance of 0.2 times SD. The detrended fluctuation analysis used a short-term parameter ranging between 4 and 12 beats and long-term fluctuations between 13 and 64 beats. Considering non-linear analysis, the DFAα1 parameter was computed. DFAα1 is considered a sensitive parasympathetic index^83^ able to discriminate possible long-term correlations and complexity of RR interval series^84^. A fractal structure of heart rate was quantified by estimating a short-term, alpha 1, fluctuations, obtained from the range 4 ≤ *n* ≤ 16. The time-varying analysis settings were: HRV analysis window width equal to 300 s with an effective data threshold at 50%. For the time-varying spectrum estimation, the well-known spectrogram was chosen.

### Statistical analysis

#### Sample size calculation

Due to lack of perfusion studies assessing the effects of manual therapies on cLBP, the computation of a priori sample size was difficult. For this reason, we computed a posteriori the effect size (Cohen’s d) for each ROI by using the baseline-T2 difference data in contrast between OMT and sham group. In addition, β values were calculated using pwr.t.test function {pwr} in R considering Cohen’s *d*, alfa = 0.05 and number of subjects equal to 16.

Arithmetic mean and standard deviation as well as median, percentage and range were used to explore the general characteristics of the study population. To compare the OMT and sham group at enrolment, univariate statistical tests, student t test and chi square test were performed.

#### HRV analysis

HRV analysis was performed in the present trial using the restricted weak stationarity (RWS) test to assess stationarity^85^, over *M* patterns. Shapiro test was applied to test the normality of R-R distribution (*p* < 0.05). In case of non-normal distribution, a log transformation was applied. Then, *M* patterns were assessed for normality. The patterns were randomly selected from a set of sequences of length *L*^85^. These patterns were then used for the final analysis.

#### Perfusion data

To study the independent effect of osteopathic manipulative treatment on perfusion data, HRV endpoints and pain data, a 2 × 3 repeated measure analysis based on MER model considering random effect for groups (OMT-SHAM) and a fixed effect for period (T0–T1–T2) was used to further explore any difference. The following confounding factors were considered in the analysis: age, gender, caffeine consumption, circadian and menstrual cycle. Post hoc pairwise analysis adjusted by Holm–Bonferroni correction was utilised after any statistical difference resulted from MER.

To indicate statistical difference, two-tailed P values of less than 0.05 was considered. This data analysis was carried out using the R statistical program (v. 3.5.2).

#### Correlation analysis between regional CBF values, VAS scores and HRV parameters

To determine whether CBF in the pain-related regions was correlated with pain intensity, Pearson correlation analyses between regional CBF values and VAS scores at the different timepoints were performed. Further Pearson-based correlation analyses were conducted exploring correlation between rCBF and HRV parameters as well as between HRV and pain.

Bonferroni correction for multiple comparison was used. In order to avoid circularity problems in the analysis^86^, ROIs were defined as spherical nodes (6 mm of radius) using independent coordinates from the literature^32,45^ (Supplementary materials—Table S2).

These ROIs showed a good spatial overlap with the pain-related regions revealed by the voxel-wise contrasts in the above analysis.

## Supplementary Information

Supplementary Figure 1.

Supplementary Figure 2.

Supplementary Information.
