# Supplementary figures and images for: Osteopathy modulates brain–heart interaction in chronic pain patients: an ASL study
Source: Sci Rep. 2021 Feb 25;11:4556. doi: 10.1038/s41598-021-83893-8 (PMC7907192; doi:10.1038/s41598-021-83893-8)

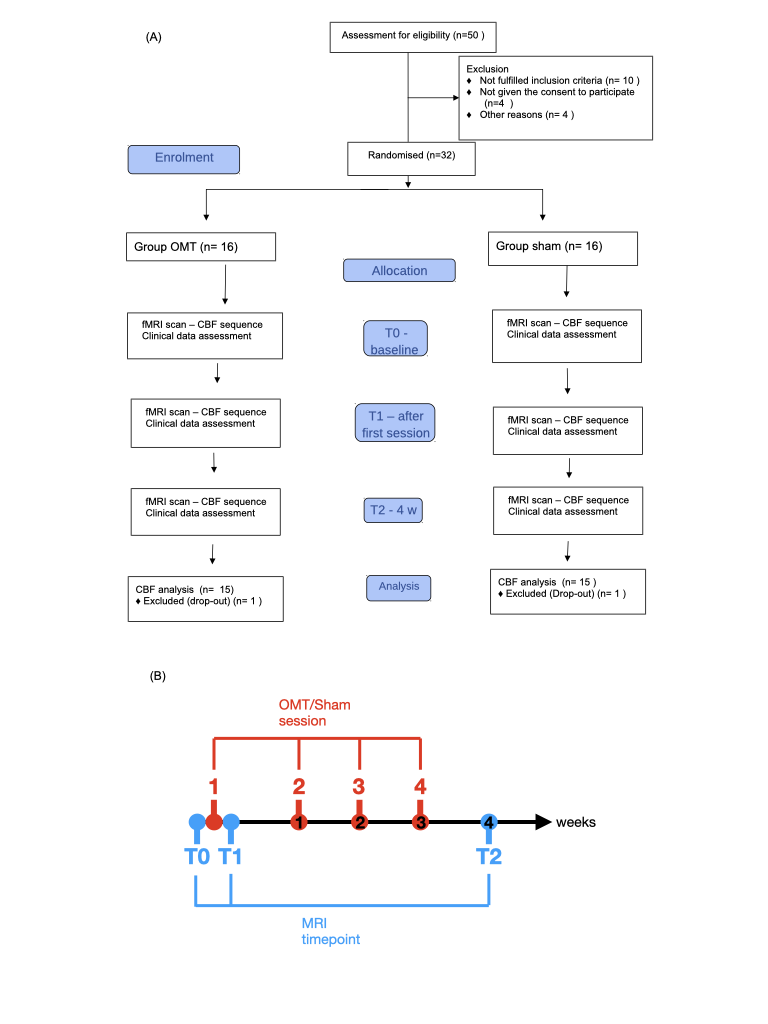

Supplement: Supplementary file 1 — Supplementary Figure 1. [file 41598_2021_83893_MOESM1_ESM.png]
