# Supplementary material for: Osteopathy modulates brain–heart interaction in chronic pain patients: an ASL study
Source: Sci Rep. 2021 Feb 25;11:4556. doi: 10.1038/s41598-021-83893-8 (PMC7907192; doi:10.1038/s41598-021-83893-8)
Supplement: Supplementary file 3 — Supplementary Information. [file 41598_2021_83893_MOESM3_ESM.docx]

**Osteopathy modulates brain-heart interaction in chronic pain patients: an ASL study**

**Francesco Cerritelli ^1,3^, Piero Chiacchiaretta ^1,2*^, Francesco Gambi ^1,2^, Raoul Saggini ^4^, Mauro Gianni Perrucci ^1,2^, Antonio Ferretti ^1,2^**

^1^Department of Neuroscience, Imaging and Clinical Sciences, “G. D’Annunzio” University of Chieti-Pescara, Chieti, Italy,

^2^ITAB-Institute for Advanced Biomedical Technologies, “G. D’Annunzio” University of Chieti-Pescara, Chieti, Italy,

^3^Clinical-Based Human Research Department— Foundation C.O.ME. Collaboration, Pescara, Italy

^4^School of Specialty in Physical and Rehabilitation Medicine, “G. D’Annunzio” University of Chieti-Pescara, Chieti, Italy

**Supplementary Materials**

**Table S1**

Mixed effect regression analysis for the brain regions of interest

| Regions | Direction of change | F values | | |
| --- | --- | --- | --- | --- |
|  |  | Group | Time | Group x Time |
| Left Posterior Insula | Decrease | 15.62^§^ | 118.76^§^ | 2.01^*^ |
| Right Ventral Anterior Insula | Increase | 6.59^#^ | 46.95^§^ | 12.50^§^ |
| Right Dorsal Anterior Insula | Increase | 0.22 | 14.89^§^ | 8.42^§^ |
| Left Anterior Cingulate Cortex | Decrease | 17.28^§^ | 80.79^§^ | 2.56^*^ |
| Left Posterior Cingulate Cortex | Increase | 16.93^§^ | 160.73^§^ | 6.87^§^ |
| Left Lentiform Nucleus | Increase | 16.47^§^ | 146.80^§^ | 6.58^§^ |
| Right Lentiform Nucleus | Increase | 10.70^§^ | 98.79^§^ | 4.59^#^ |
| Left Thalamus | Decrease | 4.85^*^ | 389.47^§^ | 0.92 |
| Right Ventroposterior Lateral Thalamic Nucleus | Increase | 13.04^§^ | 48.69^§^ | 16.16^§^ |
| Left Middle Frontal Lobe | Decrease | 1.00 | 15.28^§^ | 2.20^*^ |
| Left Cuneus | Decrease | 4.07^*^ | 123.49^§^ | 1.28 |
| Left Superior Parietal Lobe | Increase | 19.54^§^ | 32.13^§^ | 2.64^*^ |
| Right Orbito Frontal Cortex | Increase | 6.42^#^ | 28.35^§^ | 5.30^§^ |
| Right Anterior Cingulate Cortex | Increase | 8.05^§^ | 38.58^§^ | 11.14^§^ |
| Right Mid Orbital Frontal Gyrus | Increase | 6.02^#^ | 21.22^§^ | 9.29^§^ |
| Left Cerebellum (Crus 1) | Increase | 36.76^#^ | 76.57^§^ | 5.01^§^ |

F values derived from mixed effect regression (MER) analysis and are reported for the main effect Group, Time and Group x Time interaction. *p<0.05; #p<0.01; §p<0.001

**Table S2**

Clusters of significant cerebral blood flow changes at the end of the study period (T2).

| Regions | Direction of change | MNI coordinates | | | K | Cohen’s d (95%ci) | β |
| --- | --- | --- | --- | --- | --- | --- | --- |
|  |  | X | Y | Z |  |  |  |
| Left Posterior Insula | Decrease | -34 | -24 | 12 | 116 | 0.92(0.12,1.73) | 0.71 |
| Right Ventral Anterior Insula | Increase | 35 | 22 | -7 | 107 | 1.93(1.02,2.83) | 0.99 |
| Right Dorsal Anterior Insula | Increase | 35 | 23 | -2 | 87 | 1.35(0.52,2.18) | 0.96 |
| Left Anterior Cingulate Cortex | Decrease | -8 | 28 | 20 | 37 | 1.29 (0.45,2.13) | 0.94 |
| Left Posterior Cingulate Cortex | Increase | -4 | -60 | 18 | 33 | 1.11(0.29,1.93) | 0.86 |
| Left Lentiform Nucleus | Increase | -24 | -2 | 5 | 68 | 1.54(0.67,2.40) | 0.98 |
| Right Lentiform Nucleus | Increase | 25 | 7 | 0 | 71 | 1.15(0.33,1.97) | 0.88 |
| Left Thalamus | Decrease | -6 | -6 | -4 | 120 | 1.01(0.51,1.52) | 0.79 |
| Right Ventroposterior Lateral Thalamic Nucleus | Increase | 12 | -20 | 0 | 147 | 1.74(0.86,2.62) | 0.99 |
| Left Middle Frontal Lobe | Decrease | -32 | 42 | 0 | 83 | 0.50(-0.27,1.27) | 0.28 |
| Left Cuneus | Decrease | -18 | -78 | 30 | 32 | 0.64(-0.14,1.42) | 0.42 |
| Left Superior Parietal Lobe | Increase | -4 | -76 | 56 | 77 | 1.23(0.40,2.07) | 0.92 |
| Right Orbito Frontal Cortex | Increase | 5 | 25 | -28 | 48 | 0.96(0.17,1.75) | 0.75 |
| Right Anterior Cingulate Cortex | Increase | 6 | 45 | 0 | 50 | 1.37(0.54,2.20) | 0.96 |
| Right mid orbital frontal gyrus | Increase | 6 | 57 | -2 | 45 | 1.16(0.35,1.97) | 0.89 |
| Left Cerebellum (crus 1) | Increase | -30 | -75 | -24 | 131 | 1.31(0.49,2.14) | 0.95 |

K = number of voxels in cluster; β values from pwr.t.test function {pwr} in R considering Cohen’s *d*, alfa=0.05 and number of subjects equal to 16.

**Table S3**

HRV parameters data for study (OMT) and control (SHAM) group among the different timepoints.

| Parameter | OMT group | | | SHAM group | | |
| --- | --- | --- | --- | --- | --- | --- |
|  | T0 | T1 | T2 | T0 | T1 | T2 |
| Heart rate | 69.1±13.8 | 61.0±7.8 | 60.3±9.6 | 70.3±12.4 | 70.6±9.5 | 71.2±7.1 |
| Frequency Domain |  |  |  |  |  |  |
| HF (n.u.) | 40.3±12.7 | 58.8±14.8 | 53.3±14.0 | 42.3±10.3 | 52.25±13.9 | 41.3±13.1 |
| LF (n.u.) | 53.3±12.9 | 42.1±14.9 | 46.6±14.0 | 54.9±10.7 | 47.5±13.9 | 57.5±13.2 |
| LF/HF | 1.4±0.6 | 0.8±0.5 | 1.0±0.6 | 1.4±0.4 | 1.1±0.5 | 1.6±0.7 |
| Time Domain |  |  |  |  |  |  |
| RMSSD | 58.1±55.2 | 95.0±89.0 | 78.6±42.1 | 60.8±63.8 | 55.2±85.3 | 59.3±43.0 |
| NN50 | 32.6±49.8 | 49.1±38.8 | 43.6±39.7 | 33.7±45.2 | 28.3±50.0 | 34.8±38.2 |
| pNN50 | 8.4±10.7 | 14.3±7.5 | 12.5±8.8 | 8.1±8.0 | 7.9±8.4 | 8.4±6.8 |
| Non-linear |  |  |  |  |  |  |
| DFA-a1 | 0.9±0.1 | 0.7±0.2 | 0.8±0.2 | 0.8±0.2 | 0.8±0.2 | 0.9±0.2 |
| Sample Entropy | 1.6±0.7 | 1.7±0.5 | 1.8±0.5 | 1.7±0.3 | 1.7±0.5 | 1.7±0.3 |

Data are mean±sd. T0=baseline; T1= After first treatment; T2=End of the study.

**Figure S1. Flowchart of the study.** (A) Consort flowchart of the steps of the study. (B) Flowchart of the treatment sessions

**Figure S2. Longitudinal CBF delta changes for additional ROIs.** The figure shows the longitudinal CBF mean change within additional regions of interest for the two groups. *statistically significant differences (p < 0.05) between groups.
